# Robotic subtotal gastrectomy with Hugo™ robotic-assisted surgery (RAS) system: first report on a case series

**DOI:** 10.1016/j.ijscr.2025.112085

**Published:** 2025-10-21

**Authors:** R. Cammarata, V. La Vaccara, A. Catamerò, R. Coppola, D. Caputo

**Affiliations:** a Operative Research Unit of General Surgery, Fondazione Policlinico Universitario Campus Bio-Medico, Rome, 00128, Italy; b Research Unit of General Surgery, Università Campus Bio-Medico di Roma, Rome, 00128, Italy

**Keywords:** Robotic gastrectomy, D2 lymphadenectomy, Hugo™ RAS system, Minimally invasive surgery, Gastric adenocarcinoma, Roux-en-Y reconstruction

## Abstract

**Introduction:**

Robotic-assisted surgery (RAS) has increasingly gained interest in gastric cancer treatment due to its enhanced precision and vision. Hugo™ RAS is a novel system designed to improve accessibility and reduce costs. However, its use in oncologic upper gastrointestinal procedures is still scarcely documented.

**Presentation of case:**

We report four consecutive cases of subtotal gastrectomy with D2 lymphadenectomy performed using Hugo™ RAS at a single institution. Patients were selected following multidisciplinary evaluation and underwent full robotic procedures with standardized port placements. Intraoperative parameters, complications, and short-term oncologic outcomes were analyzed.

**Discussion:**

All procedures were completed successfully without conversion or major complications. Median docking and console times were within acceptable limits, though longer than traditional laparoscopy due to system learning curve and intraoperative frozen section use. Lymphadenectomy was adequate in all D2 cases with R0 resection achieved. No blood transfusions were needed. Postoperative complications included delayed gastric emptying and a duodenal stump leak. The system proved safe, and the procedural setup did not require modifications from laparoscopic standards.

**Conclusion:**

Subtotal gastrectomy with D2 lymphadenectomy using Hugo™ RAS is feasible and safe. The standardized setup and available instruments support oncologic adequacy without energy devices. Further studies are needed to validate long-term outcomes and assess comparative advantages over conventional approaches.

## Highlights

• First report on subtotal gastrectomy using Hugo™ RAS in gastric cancer
• Standardized port setup enabled safe and reproducible procedures
• Effective Roux-en-Y reconstruction achieved without energy devices
• Hugo™ RAS supported adequate lymphadenectomy and R0 resections
• Short-term outcomes suggest feasibility in upper GI oncologic surgery

## 1 Introduction

Gastric cancer remains one of the leading causes of cancer-related mortality worldwide, with surgical resection representing the cornerstone of curative treatment for localized disease. [1] Minimally invasive approaches, particularly laparoscopic gastrectomy, have become increasingly established due to their favorable short-term outcomes, while maintaining oncological adequacy. In recent years, robotic-assisted surgery (RAS) has emerged as a potential evolution in minimally invasive gastric surgery, offering enhanced dexterity, superior three-dimensional visualization, and greater surgical precision. [2] The Hugo™ RAS system (Medtronic Inc.) is a novel robotic platform introduced with the goal of expanding access to robotic surgery by addressing some of the cost and logistic limitations associated with previous systems, and the first surgical procedures have been performed in urological and gynecological fields in 2021. [3] Since its introduction, the application of this technology has expanded in terms of numbers of procedures and surgical fields and in 2023, General Surgery procedures such as cholecystectomy, gastric bypass, colectomies, abdominal wall repairs have been reported. [4,5,6,7,8] These experiences demonstrated Hugo™ RAS system to be safe and useful with satisfactory outcomes both in terms of surgical precision and oncological outcomes. Nonetheless, the lower costs of this technology, when compared to competitors, allowed us to identify in Hugo™ RAS a possible valid alternative for robotic minimally invasive oncologic and non-oncologic surgery. [9] However, its application to complex oncologic procedures, such as gastrectomy with D2 lymphadenectomy for gastric cancer, remains scarcely reported in the literature. Early institutional experiences and preliminary reports have demonstrated promising technical feasibility and safety, but comprehensive data in gastric oncologic surgery are still limited. Given the paucity of data, we aimed to describe our initial institutional experience with Hugo™ RAS for subtotal gastrectomy with D2 lymphadenectomy in patients with gastric cancer. This case series provides a detailed technical description of the procedure, perioperative outcomes, and short-term oncological results, contributing novel insights into the feasibility of this emerging platform in upper gastro-intestinal oncologic surgery. This case series has been reported in line with the PROCESS Guideline. [10] Artificial Intelligence (AI) wasn't used in the research and manuscript development.

## 2 Methods

### 2.1 Case presentations

The first case involved a 75-year-old man diagnosed with adenocarcinoma of the pylorus. His American Society of Anesthesiologists (ASA) physical status classification was 3, and his Body Mass Index (BMI) was 27.76. His past medical history included arterial hypertension and chronic atrial fibrillation. The second case concerned a 50-year-old man affected by a neuroendocrine tumor of the gastric antrum. He had an ASA score of 1 and a BMI of 22.72. No relevant past medical history was recorded. The third case involved an 83-year-old man diagnosed with gastric adenocarcinoma of the body of the stomach. He was metastatic at presentation, with liver involvement. Surgery was performed due to active gastric bleeding unresponsive to conservative and endoscopic treatments, following a decision made within a multidisciplinary team discussion. His ASA score was 3, and his BMI was 24.21. His medical history included psoriasis and arthritis. The fourth case was a 61-year-old man diagnosed with adenocarcinoma of the gastric antrum. His ASA score was 3, and his BMI was 19.61. His medical history was notable for Human Immunodeficiency Virus (HIV) positivity with undetectable viremia. Demographic and clinical characteristics of the case series are summarized in Table 1.

**Table 1:** Demographic, clinical and histological characteristics; BMI (Body Mass Index); ASA (American Society of Anesthesiologists) score.

Parameter	Case 1	Case 2	Case 3	Case 4
Procedure type	Subtotal gastrectomy + D2 lymphadenectomy	Subtotal gastrectomy + D2 lymphadenectomy	Subtotal gastrectomy	Subtotal gastrectomy + D2 lymphadenectomy
Age	75	50	83	61
Sex	Male	Male	Male	Male
BMI	27.76	22.72	24.21	19.61
ASA score	III	I	III	III
Comorbidities	Atrial fibrillation	None	None	HIV
Histology	Adenocarcinoma	Neuroendocrine tumor	Adenocarcinoma	Adenocarcinoma
Localization	Pylorus	Antrum	Body	Antrum
Clinical TNM stage	T3N0M0	T2N0M0	T4N1M1	T3N1M0
Clavien Dindo	I	0	0	II

### 2.2 Preoperative preparations and requirements

As previously reported all the surgical team members (operating surgeon, bed assistant and scrub nurse) completed the official technical training on Hugo™ RAS technology provided by the vendor company and performed the operation at Fondazione Policlinico Universitario Campus Bio-Medico of Rome. [4] We considered patients undergoing full robotic subtotal gastrectomy for a histologically proven gastric carcinoma or neuroendocrine tumor of the antrum or the body of the stomach, with pre-operative computed tomography scan (CT scan) staging and multidisciplinary team evaluation and procedure performed by the senior surgeon. We excluded laparoscopic conversion of the procedure and patients with tumors located at the gastro-esophageal junction. Written informed consent was obtained from all patients for the procedure and for the publication of anonymized clinical data and images. Demographic and clinical data (age, gender, BMI, ASA score and previous medical history etc.) of the four patients have been collected. Data regarding the operating times, including patient's entry into the operating room, docking time (defined as the time elapsed between the positioning of the robotic tower at the operating table and the completion of the connection of all robotic arms to the trocars, allowing the console surgeon to start the procedure), console time and patient exit from the operating room were analyzed. All patients underwent an upper gastrointestinal tract X-ray on postoperative day one. To assess the integrity of the gastro-jejunal anastomosis before proceeding with oral re-feeding. Postoperative course and short-term oncological outcomes have also been analyzed.

### 2.3 Step-by-step description

**Fig. 1:**
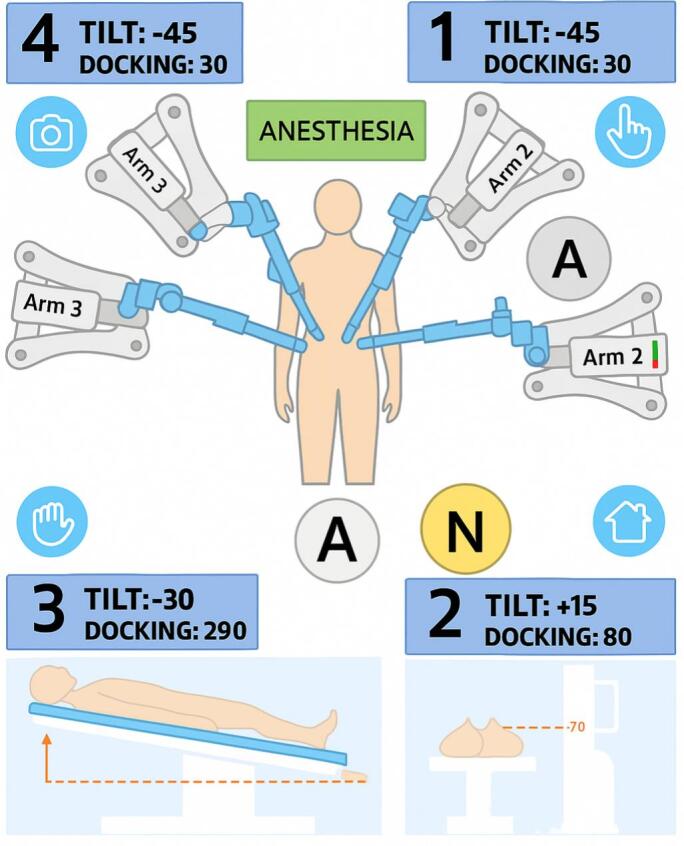
Operating room setup and tilt and docking angles. N, Scrub nurse; A, Bed assistant.

Layout of Hugo RAS™ system, consisting of the tower with the surgical console and four robotic arms, and of the operating room are shown in Fig. 1. All the procedures started with the patient in supine position with abducted legs in order to leave enough space for the bed-assistant. The first 11 mm periumbilical robotic port was placed using the open technique. Then CO2 pneumoperitoneum was induced at pressure values of 12 mmHg and an explorative laparoscopy was performed using the camera of the robotic system without docking. Once the entire abdominal cavity has been explored and the absence of further pathological findings confirmed, the table was tilted in 30° anti-Trendelenburg position. Three 8 mm robotic ports were placed after the identification of the main body prominences (costal arches and anterosuperior iliac spines) considering the 11 mm periumbilical port as the central landmark. An additional 11 mm port was placed in the left hypogastrium for the bed assistant. The position of the above-mentioned trocars was as follows:

• Right mesogastrium, on the same line of the umbilical trocar, distant about 11 cm from it.
• Left mesogastrium, on the same line of the optical trocar, distant about 11 cm from it.
• Left flank, about 10 cm from the trocar placed in the left mesogastrium, on the same line.

**Fig. 2:**
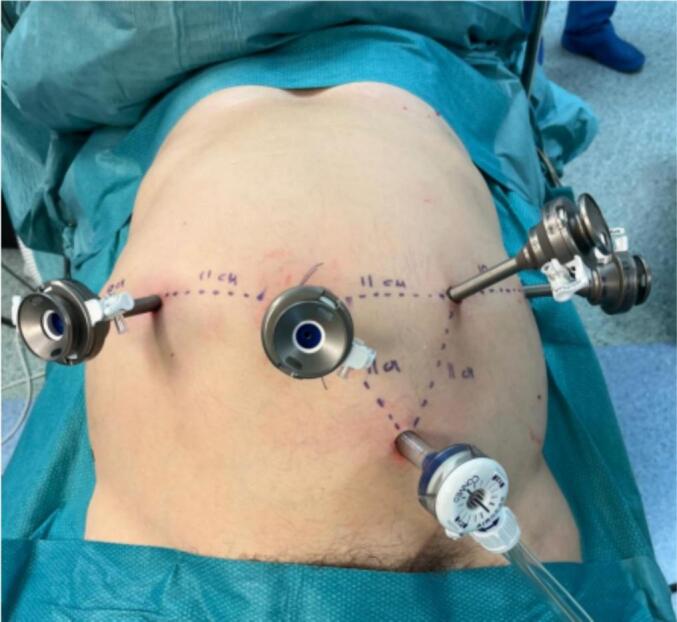
Port placement used for all the cases. The distance between the ports always respects the minimum distance required between the trocars (8 cm).

The ports sites and arms position are shown in Fig. 2. After the docking of the robotic system, bipolar forceps were inserted in the right mesogastrium port, monopolar scissors in the left mesogastrium one and a cadier forceps in the left flank port. Robotic arms position, tilt and docking angles and used instruments are summarized in the Table 2.

**Table 2:** Tilt and docking angles of the four robotic arms and instruments used.

ARM	TILT (ANGLE)	DOCKING (ANGLE)	INSTRUMENT
ARM. 1	−45°	30°	Monopolar scissors
ARM. 2	+15°	80°	Cadiere forceps
ARM. 3	−30°	290°	Bipolar forceps
ARM. 4	−45°	340°	Endoscopy

All surgeries were successfully completed. The predetermined setup provided by the vendor allowed all procedures to be completed without any high-intensity errors. The docking and tilt angles resulted optimal throughout the course of the procedures, and no conflicts occurred between the robotic arms. All surgeries were performed safely. The docking time was 8 min for the first case,12 min for second and third ones and 5 min for the fourth. Considering the preparation of the operating theater, from the entry of the patient to the start of the console time, 48 min were needed for the first patient, 74 min for the second, 78 and 45 min respectively for the third and the fourth one. The console time was 447 min for the first case. Five-hundred eighty-nine minutes were needed to complete the surgery for the second case, while 512 min was the console operative time for the third case. The fourth case console time was 548 min. In all the series the surgical procedures started with the section of the colo-epiploic and gastrocolic ligaments using the monopolar scissor allowing the access to the omental bursa. The right gastro-epiploic, and right and left gastric vessels were sectioned between hem-o-lock applied by the bed assistant surgeon using the hypogastrium accessory port. The first duodenal portion was sectioned using a single 60 mm purple charge of linear automatic stapler without Kocher's maneuver. Lymphadenectomy of the hepatic pedicle, of the common hepatic artery and the celiac trunk was performed using the bipolar forceps and the monopolar scissors in all cases except for the third one. In this case a D1 lymphadenectomy was performed. All D2 lymphadenectomies performed in this series were carried out according to internationally accepted standards, and the number of retrieved lymph nodes was consistent with the threshold recommended by both European Society For Medical Oncology (ESMO) and the Japanese Gastric Cancer Association (≥15 nodes). [11,12] The section of the left gastric artery was performed at its origin in all cases except the fourth one because of the presence of an accessory left hepatic artery arising from the left gastric artery. In this last case, the origin of the left gastric artery was dissected to achieve a complete lymphadenectomy, but the vessel was sectioned distal to the emergency of the accessory hepatic artery. The left gastroepiploic vessels were divided at the level of the gastric fundus, which was transected using multiple 60 mm purple cartridges of the powered surgical stapler. Once the specimen was retrieved via a suprapubic mini-laparotomy, the proximal section margin was sent for frozen section examination to confirm the absence of residual tumor before the transmesocolic Y-loop reconstruction. The gastric stump was reinforced with two running sutures of Stratafix 3.0. The anastomoses (gastro-jejunal and jejunum-jejunal) were performed with a 60 mm and 45 mm purple charge of stapler, respectively. Enterotomies were closed in double layers (single stitches for the interior and running suture for the exterior) in both cases. Hemostasis was checked, two drains were placed to drain duodenal stump a gastro-jejunal anastomosis, and ports were removed. Estimated intraoperative blood loss was minimal in all four procedures. Specifically, blood loss was approximately 100 mL in Case 1, 50 mL in Case 2, 80 mL in Case 3, and 70 mL in Case 4. No patient required transfusion. These values were recorded based on intraoperative suction volume and gauze count. All patients underwent an upper gastrointestinal tract X-ray on postoperative day one to assess the integrity of the gastrojejunal anastomosis before initiating oral refeeding; in all cases, the exam showed no evidence of leakage or other complications. Postoperative and oncological short-term outcomes are summarized in Table 3.

**Table 3:** Post-operative and oncological short-term outcomes.

Parameter	Case 1	Case 2	Case 3	Case 4
Procedure type	Subtotal gastrectomy + D2 lymphadenectomy	Subtotal gastrectomy + D2 lymphadenectomy	Subtotal gastrectomy + D1 lymphadenectomy	Subtotal gastrectomy + D2 lymphadenectomy
Docking time (minutes)	8	12	12	5
Console time (minutes)	447	589	512	548
Total operative time (minutes)	518	637	568	648
Intraoperative complications	None	None	None	None
Blood loss (mL)	100	50	80	70
Post-operative complications	Delayed gastric emptying	No	No	Delayed gastric emptying Duodenal stump leak
Length of stay	24 days	7 days	8 days	27 days
Final pathological staging	pT3N0, G2	NET (Ki67 8 %)	pT4aN2, G3	pT3N3a, G3
Harvested nodes	30	34	16	27
Positives nodes	0	0	4	10
Resection margins	R0	R0	R0	R0

## 3 Discussion

Robotic gastric cancer surgery gained interest with the spread of robotic surgery. Recently, Susumu Shibasaki reported how this kind of surgery can be considered in all the cases that fulfill the indication of laparoscopic gastric surgery at institutions that meet specific criteria and are approved to claim the National Health Insurance costs for the use of the surgical robot in Japan. [13] This statement raises two relevant questions; the first question concerns the usefulness of robotic surgery, the second regards the problem of its costs in gastric cancer treatment. Furthermore, starting from the assumption that the data available in literature has not established a clear advantage of robotics compared to the classic laparoscopic approach, the problem of cost becomes even more relevant and according to Kim represents the main issue that needs to be solved for the spreading of this type of surgery. [14] To date, the da Vinci surgical system dominates the robotic surgery market, but being its use is still expensive, this may represent one of the main limitations of robotic surgery when compared to laparoscopy. [15] The Authors previously gained substantial experience with robotic gastrectomy using the da Vinci surgical system, reporting favorable outcomes in terms of perioperative safety, effectiveness, and oncological adequacy, especially in elderly patients, thus supporting the rationale for exploring alternative robotic platforms such as Hugo™ RAS. [16] Hugo™ RAS has been recently introduced with the main aim of making robotics in surgery cheaper and therefore more widespread. A recent cost analysis using time-driven activity-based costing showed that, in urologic surgery, the Hugo™ system was associated with significantly lower overall costs compared to the da Vinci platform (€3511.73 vs. €4979.21), mainly due to differences in kit and platform-related expenses. [17] However, these results come from a urologic setting, and to date no similarly detailed cost analyses are available for gastrectomy. In the field of gastric surgery, the main experiences reported with Hugo™ RAS concern the treatment of bariatric patients or those affected by benign pathologies. [5,18] To date, the only gastrectomy with D2 lymphadenectomy described in literature is the one reported by Gangemi. In this paper good surgical results in terms of post-operative complications and number of harvested lymph nodes have been described. [19] To the best of our knowledge, here we report the very first small case series of consecutive subtotal gastrectomies with the Hugo™ RAS system. Compared to Gangemi, who described a subtotal gastrectomy with Billroth II reconstruction, in this present series we demonstrated that Hugo™ RAS proved to be safe and effective in performing also a Roux-en-Y reconstruction. Nevertheless, while based on previous experiences, we reported that the lack of robotic energy devices represents a main issue in the use of this robotic system in colorectal surgery, where hybrid operations (robotic-laparoscopic) to achieve dissection with complete and safe hemostasis were sometimes needed, the use of Hugo™ RAS system in gastric cancer surgery induced us to reconsider this position. [7] The combination of the activity of bipolar forceps in the surgeon's “left hand” and the monopolar scissors in the “right one”, together with the absence of the need to move into different districts of the abdominal cavity (e.g. from the gastrocolic ligament to the pelvis during a rectal resection), allowing always the use of the fourth arm for traction, guarantee optimal dissection with satisfactory control of the hemostasis without the need of other energy devices. So, from this initial experience, it's our opinion that using the already available tools is sufficient to complete a subtotal gastrectomy in an effective and safe way. In disagreement with what was experienced for colorectal surgery and for cholecystectomy [2,4], we also noticed that the setup of the robotic ports and patient on the operating table proposed by the vendor to perform the gastrectomy not only did not need to be modified for our needs but reproduce those generally used for laparoscopic surgery. [4,7]

In our opinion, this aspect could represent a strong point in case of need of conversion to the laparoscopic approach. Operative time was influenced by the learning curve associated with the adoption of a new robotic system, despite the team's previous experience with robotic and laparoscopic gastrectomies. In addition, the routine use of intraoperative frozen section analysis contributed to procedural duration. After having performed only 4 cases, we are aware that it is early to draw definitive conclusions about the effectiveness of this robotic platform. However, we feel confident in saying that our impression is that, compared to laparoscopic surgery, the lymphadenectomy and sutures performed with Hugo are favored by improved vision, greater precision and ease of execution for the operator. The results that the pathologist reported for the lymphadenectomy further demonstrate that the type of sampling is appropriate for the type of intervention. While patients in whom 30 lymph nodes were retrieved have received a classic D2 lymphadenectomy, in the patient with 16 lymph nodes harvested, a D1 was performed as the patient was already metastatic at the liver and the indication for gastrectomy was dictated by the gastric bleeding. As regards the postoperative course, the narrowness of the series does not allow us to draw definitive conclusions about this aspect; our impression is that the course of a robotic subtotal gastrectomy with Hugo™ RAS is comparable to that of a laparoscopic subtotal gastrectomy. Delay of gastric emptying represents our main complication (50 % of the cases) and influenced the length of the hospital stay together with the need of prolonged antibiotic therapy needed in one case for the treatment of an abdominal collection consequent to a mild duodenal stump leak. As stated before, if compared to laparoscopy, cost represents one of the Achilles' heels of robotics, and Hugo™ RAS has entered the market with the aim of mitigate this aspect. We absolutely cannot say that in our experience this platform allows also to overcome the other well-known limitation of robotic gastric surgery, i.e. that of the long duration of the procedures. [13] When compared with the report by Gangemi, our operative times are longer, but we believe that the difference in the reconstruction (Y-shaped vs. BII) and the time needed for frozen section examination may partly explain this difference. Furthermore, we also trust that the future availability of integrated energy tools together with greater practice will help to improve these performances. [19]

## 4 Conclusions

The Hugo™ RAS can be used to perform subtotal gastrectomy with D2 lymphadenectomy in gastric cancer patients. The predetermined setup proposed by the vendor company and the tools provided allow us to complete this type of surgery safely and with satisfying short term oncological results. Due to the limited number of cases, this report should be interpreted as a preliminary experience. Further prospective studies including larger patient cohorts, long-term follow-up, and direct comparisons with other minimally invasive approaches are needed to confirm the safety, feasibility, and potential advantages of the Hugo™ RAS system in gastric cancer surgery. This series, however, could represent the starting point to encourage the use of this new platform in the field of upper GI oncologic surgery.

## Abbreviations

**Table:** 

ASA	American Society of Anesthesiologists
BMI	Body Mass Index
CT	Computed Tomography
D1	First-tier lymphadenectomy
D2	Second-tier lymphadenectomy
GI	Gastrointestinal
HUGO™ RAS	Hugo™ Robotic-Assisted Surgery system
OR	Operating Room
R0	Complete macroscopic and microscopic resection
TNM	Tumor-Node-Metastasis classification
X-ray	Radiographic imaging

## Consent

The patient signed an informed consent form prior to surgery, acknowledging the procedure, associated risks, and alternatives. Consent was obtained according to institutional and ethical standards.

## Ethical approval

Ethical approval was not required for this case series

## Funding

This research did not receive any specific grant from funding agencies in the public, commercial, or not-for-profit sectors.

## Author contribution

- Conceptualization: Roberto Cammarata, Vincenzo La Vaccara, Alberto Catamerò, Damiano Caputo, Roberto Coppola
- Data curation: Roberto Cammarata, Vincenzo La Vaccara, Alberto Catamerò, Damiano Caputo, Roberto Coppola
- Formal analysis: Roberto Cammarata, Vincenzo La Vaccara, Alberto Catamerò, Damiano Caputo, Roberto Coppola
- Funding acquisition: Roberto Cammarata, Vincenzo La Vaccara, Alberto Catamerò, Damiano Caputo, Roberto Coppola
- Investigation: Roberto Cammarata, Vincenzo La Vaccara, Alberto Catamerò, Damiano Caputo, Roberto Coppola
- Methodology: Roberto Cammarata, Vincenzo La Vaccara, Alberto Catamerò, Damiano Caputo, Roberto Coppola
- Project administration: Damiano Caputo, Roberto Coppola
- Resources: Roberto Cammarata, Vincenzo La Vaccara, Alberto Catamerò, Damiano Caputo, Roberto Coppola
- Software: Roberto Cammarata, Vincenzo La Vaccara, Alberto Catamerò, Damiano Caputo, Roberto Coppola
- Supervision: Damiano Caputo, Roberto Coppola
- Validation: Damiano Caputo, Roberto Coppola
- Visualization: Roberto Cammarata, Vincenzo La Vaccara, Alberto Catamerò, Damiano Caputo, Roberto Coppola
- Writing – original draft: Roberto Cammarata, Vincenzo La Vaccara, Alberto Catamerò, Damiano Caputo, Roberto Coppola
- Writing – review & editing: Damiano Caputo, Roberto Coppola

## Guarantor

Professor Damiano Caputo is the guarantor of the work.

## Research registration number

Not applicable.

## Conflict of interest statement

The authors declare that they have no conflict of interest regarding the publication of this case series.
